# Accuracy of Genomic Selection for Important Economic Traits of Cashmere and Meat Goats Assessed by Simulation Study

**DOI:** 10.3389/fvets.2022.770539

**Published:** 2022-03-16

**Authors:** Xiaochun Yan, Tao Zhang, Lichun Liu, Yongsheng Yu, Guang Yang, Yaqian Han, Gao Gong, Fenghong Wang, Lei Zhang, Hongfu Liu, Wenze Li, Xiaomin Yan, Haoyu Mao, Yaming Li, Chen Du, Jinquan Li, Yanjun Zhang, Ruijun Wang, Qi Lv, Zhixin Wang, Jiaxin Zhang, Zhihong Liu, Zhiying Wang, Rui Su

**Affiliations:** ^1^College of Animal Science, Inner Mongolia Agricultural University, Hohhot, China; ^2^Inner Mongolia Bigvet Co., Ltd., Hohhot, China; ^3^College of Veterinary Medicine, Inner Mongolia Agricultural University, Hohhot, China; ^4^Department of Obstetrics and Gynaecology, Inner Mongolia Medical University, Hohhot, China; ^5^Key Laboratory of Mutton Sheep Genetics and Breeding, Ministry of Agriculture, Hohhot, China; ^6^Key Laboratory of Animal Genetics, Breeding and Reproduction in Inner Mongolia Autonomous Region, Hohhot, China; ^7^Engineering Research Centre for Goat Genetics and Breeding, Inner Mongolia Autonomous Region, Hohhot, China

**Keywords:** genomic selection, marker density panel, reference population, number of QTLs, goats

## Abstract

Genomic selection in plants and animals has become a standard tool for breeding because of the advantages of high accuracy and short generation intervals. Implementation of this technology is hindered by the high cost of genotyping and other factors. The aim of this study was to determine an optional marker density panel and reference population size for using genomic selection of goats, with speculation on the number of QTLs that affect the important economic traits of goats. In addition, the effect of buck population size in the reference population on the accuracy of genomic estimated breeding value (GEBV) was discussed. Based on the previous genetic evaluation results of Inner Mongolia White Cashmere Goats, live body weight (LBW, *h*^2^ = 0.11) and fiber diameter (FD, *h*^2^ = 0.34) were chosen to perform genomic selection in this study. Reasonable genome parameters and generation transmission processes were set, and phenotypic and genotype data of the two traits were simulated. Then, different sizes of the reference population and validation population were selected from progeny. The GEBVs were obtained by six methods, including GBLUP (Genomic Best Linear Unbiased Prediction), ssGBLUP (Single Step Genomic Best Linear Unbiased Prediction), BayesA, BayesB, Bayesian ridge regression, and Bayesian LASSO. The correlation coefficient between the predicted and realized phenotypes from simulation was calculated and used as a measure of the accuracy of GEBV in each trait. The results showed that the medium marker density Panel (45 K) could be used for genomic selection in goats, which can ensure the accuracy of the GEBV. The reference population size of 1,500 can achieve greater genetic progress in genomic selection for fiber diameter and live body weight in goats by comparing with the population size below this level. The accuracy of the GEBV for live body weight and fiber diameter was better when the number of QTLs was 100 and 50, respectively. Additionally, the accuracy of GEBV was discovered to be good when the buck population size was up to 200. Meanwhile, the accuracy of the GEBV for medium heritability traits (FDs) was found to be higher than the accuracy of the GEBV for low heritability traits (LBWs). These findings will provide theoretical guidance for genomic selection in goats by using real data.

## Introduction

As one of the earliest domesticated species, goats are distributed mainly in remote areas of some countries, including China, Mongolia, Australia, India, Iran, Pakistan, and New Zealand. Because of advantages of cashmere yield, fiber diameter, and body weight, Chinese cashmere goats are well-known worldwide. Inner Mongolia cashmere goats and Liaoning cashmere goats are widely used as paternal lines of other breeds of goats in China. Both breeds are prohibited from being exported abroad. Breeders in China have begun to perform selection for cashmere goats in 1980s. At first, it was selected according to cashmere color, then it was selected by phenotype records, and the estimated breeding value selection was implemented in 1998. Up to now, the selection of superior goats was based on estimated breeding value for liveweight and fleece traits in a large number of cashmere goat breeds (1–3). The increasing trend observed in the Chinese goat cashmere yield from 2001 to 2018 years reflects an increasing economic importance.

The Chinese goat population size is gradually decreasing, from 15.2 million in 2004 to 13.8 million in 2019. However, the cashmere yield increased first and then was kept stable from 2004 to 2019. Therefore, it is necessary to use more advanced breeding methods to improve the production performance of goats.

The idea of genomic selection was proposed and published by (4), enabling selection decisions to be made early in the life of animals. This approach is beneficial for traits that are difficult to measure and traits with low heritability. This method has been successfully applied to other livestock species, such as dairy cattle, beef, pigs, chickens, and sheep (5–9). Due to the limited marginal economic value of a goat breeding system, to the substantial number of markers required for genomic selection and to the high cost of sequencing, genomic selection in goats is still limited. In recent years, breeding programs based on genomic selection have been developed in dairy goats in France and the UK (10, 11), but the reference population size is relatively small. To date, genomic selection of cashmere goats has not been reported yet.

Meuwissen and Goddard used whole genome sequence data for the prediction of the genetic values of individuals for complex traits and obtained a prediction accuracy higher than 0.80 (12). The accuracy of genomic predictions is affected by many factors, including marker density, the level of linkage disequilibrium (LD) between the markers and QTLs, reference population size, heritability of the trait, and distribution of QTLs and GEBV methods (13–15). Muir illustrated that the increase of marker density and phenotype information can improve accuracy of genomic selection (16). The average variance proportion of each QTL decreases with the increase in number of QTLs. When the number of QTLs is greater, it is more difficult to accurately estimate the effect of markers around each QTL, which can lead to an increase in estimation error rate. Ma et al. showed that QTL markers improved the reliability of genomic prediction. Additionally, this study illustrated that the reference population including bulls that have more progeny can increase GEBV predicted accuracy (17). Lillehammer et al. used simulated data to perform genomic selection of maternal traits in pigs, which illustrated that the genetic progress obtained by the population size of 1,000 was found to be 75% of the genetic progress of 5,000 (18). Anna Wolc et al. used simulated data to perform genomic selection of laying chickens, and found that the generation interval was shortened by half (19). Villumsen et al. used simulated data to perform genomic selection, which demonstrated that accuracy evaluation of genomic breeding value improved nearly 17% when the heritability increases from 0.02 to 0.30 (20). Clark et al. compared the accuracy of GEBV by using BLUP, GBLUP, and BayesB methods, which illustrated that the prediction will be more accurate by using BayesB if some important QTLs existed, no significant difference between GBLUP and BayesB was observed when the QTL effect was small (21).

Although the genomic selection has been well-applied to other breeding animals, including cattle, pigs, and chicken, it has been proved to obtain better selection accuracy. But the genomic selection of Chinese goats has not been reported yet. The aim of this study was to evaluate the potential effect of the density of marker panels, reference population sizes, number of QTLs, prediction methods, and buck population size in a reference population on the accuracy of GEBV for the important economic traits of goats.

## Materials and Methods

Records of 7,102 animals collected from 1988 to 2000 at the Inner Mongolia White Cashmere goat breeding farm were used by Zhou (1) to estimate genetic parameters of Cashmere fiber diameter and live body weight. The results showed that their heritability ranged from medium (0.34 for fiber diameter) to low (0.11 for body weight). Based on these findings, both traits were used as example traits in a successive simulation analysis. Combining the genomic sequence information in goats published in NCBI (https://www.ncbi.nlm.nih.gov/genome/?term=goats), QMSim software was used to produce phenotype and genotype data by simulation. Then, GEBV for both traits was obtained with BGLR and HIBLUP packages in R (22). Then the related factors affected accuracy of GEBV was evaluated by correlation coefficient between the predicted and realized phenotypes from simulation.

### Data Simulation

Using QMSim software (23), the populations were simulated based on a forward in-time process (24). In the first simulation step, 5,000 generations with a constant size of 1,000 (500 males and 500 females) were simulated, followed by 500 generations with a gradual increase in population size from 1,000 to 3,000 (400 males and 2,600 females) to create initial LD and establish mutation-drift equilibrium in historical generations. In the second step, an expansion of the population was created by initially randomly selecting 40 founder males and 400 founder females from the last generation of the historical population. To enlarge the population, 10 generations were simulated with 5 offspring per dam. The mating system was based on the random union of gametes with no selection. Subsequently, 40 males and 400 females from the last generation of the expanded population were randomly mated to generate another 10 generations with 15, 30, 45, and 60 K SNP markers, respectively. The parameters used in recent generations mimicked a real production system with one or two progeny per dam per year, 50% of male progeny, selection for high values of EBV in live body weight (*LBW, h*^2^ = 0.11) and low values of EBV in fiber diameter (*FD, h*^2^ = 0.34), then culling for individuals with a replacement rate of 80% for sires and 30% for dams. Sires and dams were randomly mated.

The simulated genome consisted of 29 pairs of autosomes with lengths identical to the real Capra hircus genome based on *de novo* assembly (https://www.ncbi.nlm.nih.gov/genome/?term=goats) (25) totaling 2,922 cM. In most reported simulation studies, only one chromosome was simulated because of the limitation of computing time and memory requirements. The advantage of simulating a real number of autosomes with lengths identical to the goat genome is to create a more realistic scenario. The SNP markers were randomly distributed, and the initial number of markers was chosen, such as 15, 30, 45, or 60 K. A total of 50, 100, and 150 QTLs were randomly distributed among the markers. The effects of QTLs were sampled from a gamma distribution with shape parameters of 0.40. The mutation rate of the markers and QTLs was assumed to be 9.4 × 10^−6^ per locus per generation. The crossover interference was set to 5.0 by referring to studies on other ruminants (26). The parameters used for simulating population structure and genomes are given in Table 1.

**Table 1:** Parameters of the simulation process.

**Population structure**	**Populations**
**Step1: Historical generations (HGs)**	
Number of generations (size)-phase 1	5,000 (1,000)
Number of generations (size)-phase 2	500 (3,000)
**Step 2: Expanded generations (EGs)**	
Number of founder males from HG	400
Number of founder females from HG	2,600
Number of generations	10
Number of offspring per dam	5
**Step3: Recent generations**	
Number of founder males from EG	40
Number of founder females from EG	400
Number of generations	10
Number of offspring per dam	1, 2
Ratio of male	50%
Mating system	Random
Replacement ratio of males	80%
Replacement ratio of females	30%
Selection	EBV
Culling	Age
BV estimation method	BLUP animal model
Heritability of the traits	0.11, 0.34
Phenotypic variance	1.0
**Genome**	
Number of chromosomes	29
Total of genome length	2922 cM
Number of markers	15,000/30,000/45,000/60,000
Marker distribution	Random
Number of marker alleles	2
Number of QTLs	50/100/150
QTL distribution	Random
Number of QTL alleles	2
QTL allele effect	Gamma distribution (shape = 0.40)
Rate of recurrent mutation	9.4*10–6
Crossover interference	5.0

*EBV, estimated breeding value; BLUP, best linear unbiased prediction; QTL, quantitative trait loci; the whole simulation was repeated 10 times*.

### Reference and Validation Sets

**Figure 1:**
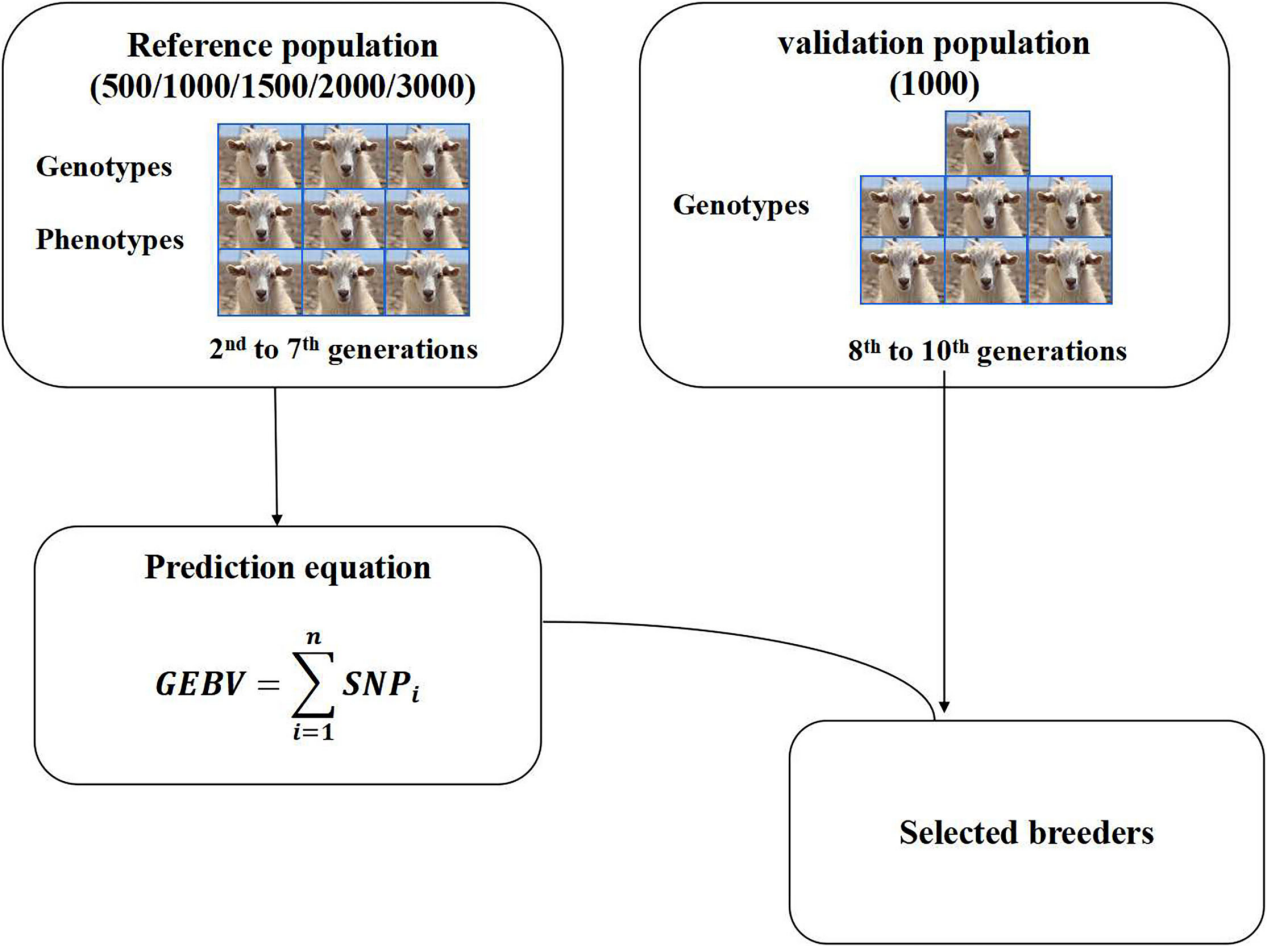
Genomic selection scheme.

Some reports have shown that reference population size has a significant effect on the accuracy of GEBV (18, 27). Five reference population sizes (500, 1,000, 1,500, 2,000, 3,000) and one validation population size 1,000 were selected to perform genomic selection in this study. The individuals in the reference population were obtained by random sampling from the 2nd to 7th generations. The individuals in the validation population were obtained by random sampling from the 8–10th generations (Figure 1). The individuals in the reference population and the validation population were selected by the random sampling method, and each population size was repeated 3 times. Reference datasets with phenotypes and genotypes were used to predict marker effects. The accuracy of genomic selection was evaluated based on the selected reference group and validation group. After qualifying the reference population size, different groups were set according to different male content in the reference population, and then the effects of different male content on the accuracy of genomic selection were studied.

### Methods of Estimating Genomic Breeding Value

Many methods have been applied to estimate genomic breeding value. However, the accuracy of GEBV among the different traits varies among the methods. Using the genotype and phenotype data produced from simulation, GEBV was obtained with GBLUP (Genomic Best Linear Unbiased Prediction), ssGBLUP (Single Step Genomic Best Linear Unbiased Prediction), and Bayesian series methods such as BayesA, BayesB, Bayesian ridge regression (BRR), and Bayesian LASSO (BL). All models were fitted using the BGLR (22) and HIBLUP (https://www.hiblup.com/) R packages.

GBLUP is a method that utilizes genomic relationships to estimate the genetic merit of an individual (28, 29). The genomic relationship matrix defines the covariance between individuals based on observed similarity at the genomic level, rather than on expected similarity based on pedigree, so that more accurate predictions of merit can be obtained. The GBLUP method assigns the same variance to all loci and essentially treats them all as equally important. However, a series of Bayesian methods give more emphasis to some genomic regions by allowing the variance to differ between SNP loci. The BayesA method assumes that the effects of all SNPs on phenotype obey the normal distribution${\;g}_{i}\sim N\left(0,\alpha_{g_{i}}^{2}\right)$, and the variance $\sigma_{g_{i}}^{2}\;$is subject to the inverse chi-square distribution $\sigma_{g_{i}}^{2}\sim \chi^{-2}\left(v,S\right)$, *v* is the degree of freedom, and *S* is the scale parameter (30). Most of the markers have very small effects on phenotype, while only a few have large effects. The distribution of genetic variances across loci is that some have no genetic variance, and a few have genetic variance. However, the prior density of BayesA does not have a density peak at $\sigma_{g_{i}}^{2}=0$. In fact, its probability of $\sigma_{g_{i}}^{2}=0$ is infinitesimal. The BayesB method uses a prior that has a high density,π, at $\sigma_{g_{i}}^{2}=0$ and has an inverted chi-square distribution for $\sigma_{g_{i}}^{2}>0.$ In the Bayesian Lasso (31), the prior assigned to marker effects is a Laplace (double exponential, DE) distribution. All marker effects are assumed to be independently and identically distributed as a DE. These priors assign the same variance or prior uncertainty to all marker effects, but they possess thicker tails than the normal or Gaussian prior. No fixed effects were considered in this study, and only additive genetic effects and standard deviation effects were included in the model. Therefore, the statistical methods of Bayes-Alphabet involved in this study can be written as:


$$y=\sum_{i=1}^{n}Z_{i}a_{i}+e$$

*y* is the phenotypic value vector of animals, *Z*_*i*_ is the design matrix of genotype at the *i*^*th*^ site, *a*_*i*_ is the effect value of the *i*^*th*^ marker, n is the number of markers. $\overset{n}{\underset{i=1}{\sum }}Z_{i}a_{i}$ is the breeding value of animals, and *e* is the vector of residual effects. The hypothetical distribution of all markers' effects in different Bayes methods and the formula of effect distribution are various.

The method of GBLUP involved in the current study was as follow:


$$\begin{array}{l} y=1_{n}\mu +Za+e \end{array}$$

*y* is the phenotype vector of animals, 1_*n*_ is a vector of ones, μ is overall mean, *Z* is a design matrix corresponding to the additive effect value, and *a* is the vector of the breeding value for an individual. The covariance matrix of additive effects is represented by $Var\left(a\right)=G\sigma_{a}^{2}$, where G is the matrix of relationships between individuals obtained from genomic information, $\sigma_{a}^{2}$ is the variance of additive genetic. e is a vector of random normal deviates.

The single-step genomic BLUP (ssGBLUP) was provided by Legarra et al. (32). The core idea of the ssGBLUP method is to combine a pedigree relationship matrix (A) and a genomic relationship matrix (G) to reconstruct a new relationship matrix (H) (33–36). Excepting the relationship matrix, the theory and method of ssGBLUP had no difference from the GBLUP method.

### Accuracy of Genomic Estimated Breeding Value

Each marker effect was estimated by using phenotype and genotype information in the reference population with the above model. Then, the GEBV for the validation population was obtained by summing the effects of all the markers carried by individuals. The phenotype for the validation populations was computed by adding GEBV and residual error effects. The correlation coefficient between the predicted and realized phenotypes from simulation was calculated and used as a measure of the accuracy of GEBV.


$$\begin{array}{l} r=\frac{Cov\left(\hat{P},P\right)}{\sigma_{\hat{P}}\sigma_{P}}\; \end{array}$$

$Cov\left(\hat{P},P\right)$ is the covariance of the predicted and realized phenotypes in the validation population, $\sigma_{\hat{P}}$ is the standard deviation of the predicted phenotype, and σ_*P*_ is the standard deviation of the realized phenotype.

Finally, a generalized linear model was used to evaluate the effect of marker density panel (15, 30, 45, and 60 K), reference population size (500, 1,000, 1,500, 2,000, 3,000), number of QTLs (50, 100, 150), and the number of males in the reference population (100 M + 1,400 F, 200 M + 1,300 F, 400 M + 1,100 F, 800 M + 700 F, 120 M + 1,400 F, 1,500 M) on the accuracy of GEBV. Duncan's test was used to compare differences between various levels in each factor. The general linear model (GLM) was established to perform a significance test with SAS software (37). Duncan's test was used to compare differences between various levels in each factor.

## Results

In this study, the effect of marker density panel, reference population size, number of QTLs, and buck population size in the reference population on accuracy of GEBV were discussed, which can guide the scientific and effective realization of genomic selection in cashmere and meat goats. Based on the previous genetic evaluation results, two important economic traits, fiber diameter (FD) and live body weight (LBW), were selected for analysis. Fiber diameter is a medium heritability trait, and live body weight is a low heritability trait.

### Variance Analysis of Factors on Accuracy of GEBV

The results of variance analysis of marker density panel, reference population size, number of QTLs, and methods is presented in Table 2. For fiber diameter, marker density panel had significant effect on accuracy of GEBV (*P*< *0.05*), other factors in this study had highly significant effect on accuracy of GEBV (*P*< *0.01*). For live body weight, all the factors in this study had highly significant effect on accuracy of GEBV (*P*< *0.01*).

**Table 2:** Variance analysis of different factors on the accuracy of GEBV for fiber diameter and live body weight.

	**Source**	**DF**	**SS**	**MS**	**F**	***P*-value**
Fiber diameter	Marker density panel	3	0.0111	0.0037	3.72	<0.05
	Number of QTLs	2	0.0418	0.0209	21.05	<0.01
	Reference population size	4	0.1693	0.0423	42.62	<0.01
	Methods	5	0.2295	0.0459	46.21	<0.01
	Error	201	0.1996	0.0010		
	Corrected Total	215	0.6512			
Live body weight	Marker density panel	3	0.1867	0.0621	36.42	<0.01
	Number of QTLs	2	0.0518	0.0259	15.20	<0.01
	Reference population size	4	0.5037	0.1259	73.92	<0.01
	Methods	5	0.6424	0.1285	75.41	<0.01
	Error	201	0.3424	0.0017		
	Corrected Total	215	1.5424			

*DF, degree of freedom; SS, St dev square; MF, mean square*.

### Effect of Marker Density Panel on Accuracy of GEBV

**Figure 2:**
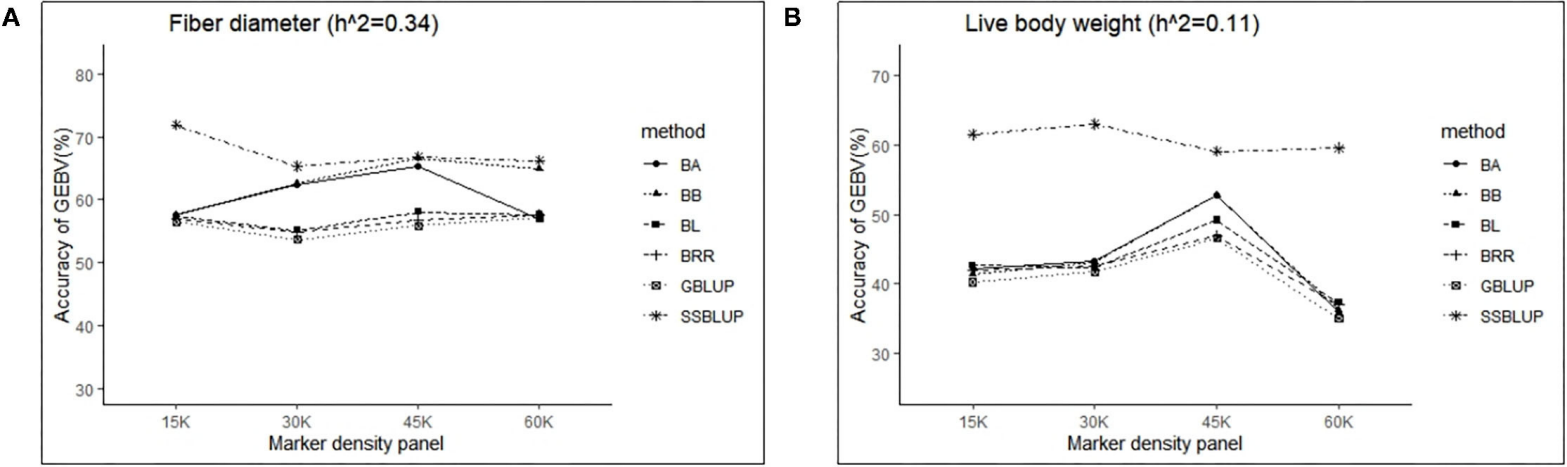
The change trends of accuracy of GEBV with increasing marker density panel with GBLUP and Bayes method.

Controlling other factors at the same level, the accuracy of GEBV in four marker density panels (15, 30, 45, and 60 K) with the GBLUP, ssGBLUP, and Bayes methods is shown in Table 3. The results obtained by GLM using the least square means method demonstrated that the marker density panel had a significant effect on the accuracy of GEBV for both traits. For FD, the accuracy of GEBV at 45 K is significantly higher than the accuracy of GEBV at 15 and 30 K. The results obtained at 45 and 60 K were not significantly different for FD. However, the accuracy of genomic selection for low heritability traits at 45 K was obviously higher than the accuracy of genomic selection at 15, 30, and 60 K. In general, an increasing trend was observed for the accuracy of GEBV with the marker density panel from 15 to 45 K in both traits (Figure 2). However, it is interesting that the accuracy of GEBV at 60 K for LBW is significantly lower than the accuracy of GEBV at 45 K (Figure 2B). Under the best marker density panel, the correlation coefficients between the predicted and realized phenotypes for the validation population with medium and low heritability traits reached 66.7 and 52.7% under the BayesB method, respectively. The accuracy of GEBV for medium heritability traits was discovered to be higher than the accuracy of GEBV for low heritability traits.

**Table 3:** Accuracy of GEBV in four marker density panels with heritability of fiber diameter and live body weight under different models.

**Methods**	**Marker density panel (*h*^2^ =** **0.34)**				**Marker density panel (*h*^2^ =** **0.11)**			
	**15 K**	**30 K**	**45 K**	**60 K**	**15 K**	**30 K**	**45 K**	**60 K**
BA	0.5754 ± 0.0069^b^	0.6233 ± 0.0134^ab^	0.6539 ± 0.0184^a^	0.5703 ± 0.0328^b^	0.4208 ± 0.0128^bc^	0.4331 ± 0.0123^b^	0.5269 ± 0.0340^a^	0.3594 ± 0.0297^c^
BB	0.5760 ± 0.0026^c^	0.6261 ± 0.0100^b^	0.6667 ± 0.0139^a^	0.6493 ± 0.0175^ab^	0.4135 ± 0.0127^b^	0.4307 ± 0.0130^b^	0.5270 ± 0.0249^a^	0.3625 ± 0.0094^c^
BL	0.5729 ± 0.0054^a^	0.5510 ± 0.0329^a^	0.5804 ± 0.0125^a^	0.5767 ± 0.0266^a^	0.4269 ± 0.0179^b^	0.4235 ± 0.0152^b^	0.4919 ± 0.0307^a^	0.3731 ± 0.0218^c^
BRR	0.5705 ± 0.0070^a^	0.5489 ± 0.0327^a^	0.5684 ± 0.0179^a^	0.5757 ± 0.0272^a^	0.4195 ± 0.0380^ab^	0.4249 ± 0.0124^ab^	0.4696 ± 0.0304^a^	0.3703 ± 0.0232^b^
GBLUP	0.5643 ± 0.0030^a^	0.5366 ± 0.0356^a^	0.5581 ± 0.0201^a^	0.5718 ± 0.0221^a^	0.4025 ± 0.0458^ab^	0.4171 ± 0.0178^ab^	0.4658 ± 0.0206^a^	0.3498 ± 0.0273^b^
ssGBLUP	0.7189 ± 0.0102^a^	0.6535 ± 0.0246^b^	0.6689 ± 0.0180^ab^	0.6623 ± 0.0309^ab^	0.6151 ± 0.0323^a^	0.6299 ± 0.0050^a^	0.5909 ± 0.0071^a^	0.5957 ± 0.0677^a^

*BA, BayesA; BB, BayesB; BL, Bayesian Lasso; BRR, Bayesian Ridge Regression; GBLUP, Genomic Best Linear Unbiased Prediction; ssGBLUP, Single Step Genomic Best Linear Unbiased Prediction*.

a, b *represent significant differences. The difference is significant with different letters*.

### Effect of Reference Population Size on Accuracy of GEBV

**Figure 3:**
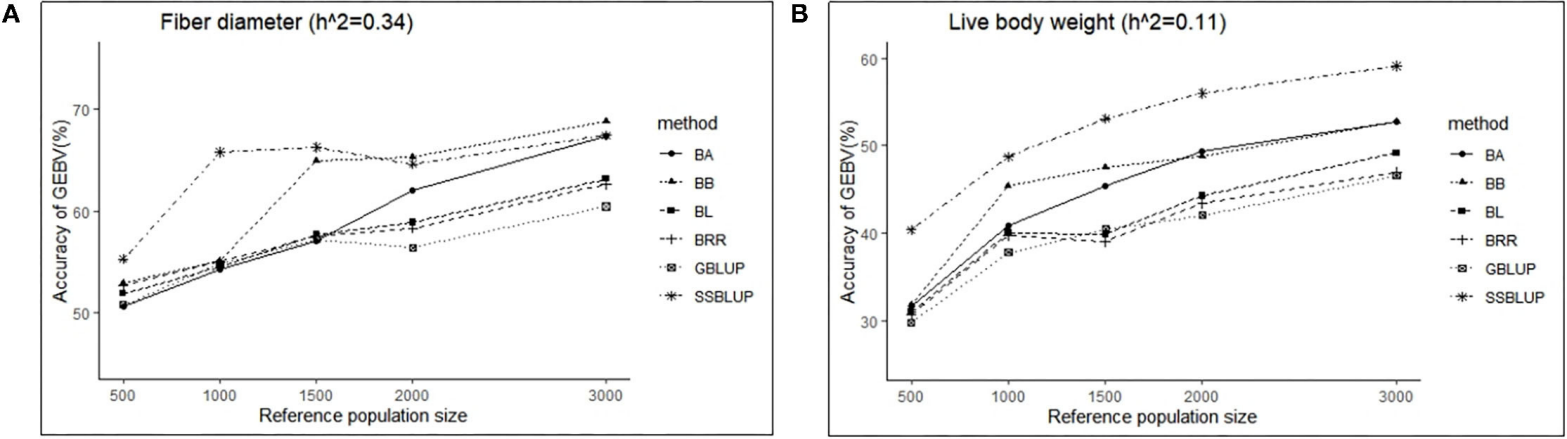
The change trends of accuracy of GEBV with increasing reference population size with GBLUP and Bayes methods.

Similarly, the effects of reference population size on the accuracy of GEBV were analyzed by controlling other factors at the same level. The results are shown in Tables 4, 5. Reference population size had a significant effect on the prediction accuracy of FD and LBW. The accuracy of GEBV with reference population sizes of 1,500, 2,000, and 3,000 was significantly higher than the accuracy of GEBV with the reference populations of 500 and 1,000 for both traits. No significant difference in prediction accuracy was discovered among the 1,500, 2,000, and 3,000 training sets. Increasing trends of GEBV accuracy were observed in FD and LBW (Figure 3). The average genomic accuracy ranged from 55.27 to 67.4% for the medium heritability trait (Table 4) and from 40.39 to 59.09% for the low heritability trait with the ssGBLUP method (Table 5). Meanwhile, the accuracy value with the ssGBLUP methods was found to be higher than the accuracy value with the GBLUP methods.

**Table 4:** Accuracy of GEBV in five reference population sizes with heritability of fiber diameter under different models.

**Methods**	**Reference population size (*h*^2^ **=** 0.34)**				
	**500**	**1,000**	**1,500**	**2,000**	**3,000**
BA	0.5061 ± 0.0351^c^	0.5427 ± 0.0545^bc^	0.5703 ± 0.0328^bc^	0.6205 ± 0.034^ab^	0.6734 ± 0.0185^a^
BB	0.5292 ± 0.0307^b^	0.5514 ± 0.0574^b^	0.6493 ± 0.0175^a^	0.6527 ± 0.0247^a^	0.6876 ± 0.0108^a^
BL	0.5190 ± 0.0378^b^	0.5455 ± 0.064^ab^	0.5767 ± 0.0266^ab^	0.5885 ± 0.0197^ab^	0.6312 ± 0.0135^a^
BRR	0.5272 ± 0.0426^b^	0.5502 ± 0.0594^b^	0.5757 ± 0.0272^ab^	0.5829 ± 0.0208^ab^	0.6262 ± 0.0108^a^
GBLUP	0.5080 ± 0.0328^b^	0.5483 ± 0.0330^ab^	0.5718 ± 0.0221^ab^	0.5641 ± 0.0190^ab^	0.6044 ± 0.0113^a^
ssGBLUP	0.5527 ± 0.0655^b^	0.6575 ± 0.0488^a^	0.6623 ± 0.0309^a^	0.6459 ± 0.0250^a^	0.6740 ± 0.0108^a^

*BA, BayesA; BB, BayesB; BL, Bayesian Lasso; BRR, Bayesian Ridge Regression; GBLUP, Genomic Best Linear Unbiased Prediction; ssGBLUP, Single Step Genomic Best Linear Unbiased Prediction*.

a, b *represent significant differences. The difference is significant with different letters*.

**Table 5:** Accuracy of GEBV in five reference population sizes with heritability of live body weight under different models.

**Methods**	**Reference population size (*h*^2^ **=** 0.11)**				
	**500**	**1,000**	**1,500**	**2,000**	**3,000**
BA	0.3171 ± 0.0870^c^	0.4082 ± 0.0321^b^	0.4543 ± 0.0344^ab^	0.4929 ± 0.0212^ab^	0.5269 ± 0.0340^a^
BB	0.3190 ± 0.0915^b^	0.4545 ± 0.0213^ab^	0.4757 ± 0.0342^ab^	0.4875 ± 0.0274^a^	0.5270 ± 0.0249^a^
BL	0.3103 ± 0.1020^b^	0.4003 ± 0.0330^ab^	0.3990 ± 0.0459^ab^	0.4427 ± 0.0153^ab^	0.4919 ± 0.0307^a^
BRR	0.3078 ± 0.1020^b^	0.3976 ± 0.0312^ab^	0.3902 ± 0.0399^ab^	0.4340 ± 0.0170^ab^	0.4696 ± 0.0304^a^
GBLUP	0.2983 ± 0.0862^b^	0.3780 ± 0.0273^ab^	0.4048 ± 0.0773^ab^	0.4204 ± 0.0246^ab^	0.4658 ± 0.0206^a^
ssGBLUP	0.4039 ± 0.0763^c^	0.4875 ± 0.0389^bc^	0.5308 ± 0.0658^ab^	0.5597 ± 0.0216^ab^	0.5909 ± 0.0071^a^

*BA, BayesA; BB, BayesB; BL, Bayesian Lasso; BRR, Bayesian Ridge Regression; GBLUP, Genomic Best Linear Unbiased Prediction; ssGBLUP, Single Step Genomic Best Linear Unbiased Prediction*.

a, b *represent significant differences. The difference is significant with different letters*.

### Effect of Number of QTLs on Accuracy of GEBV

**Figure 4:**
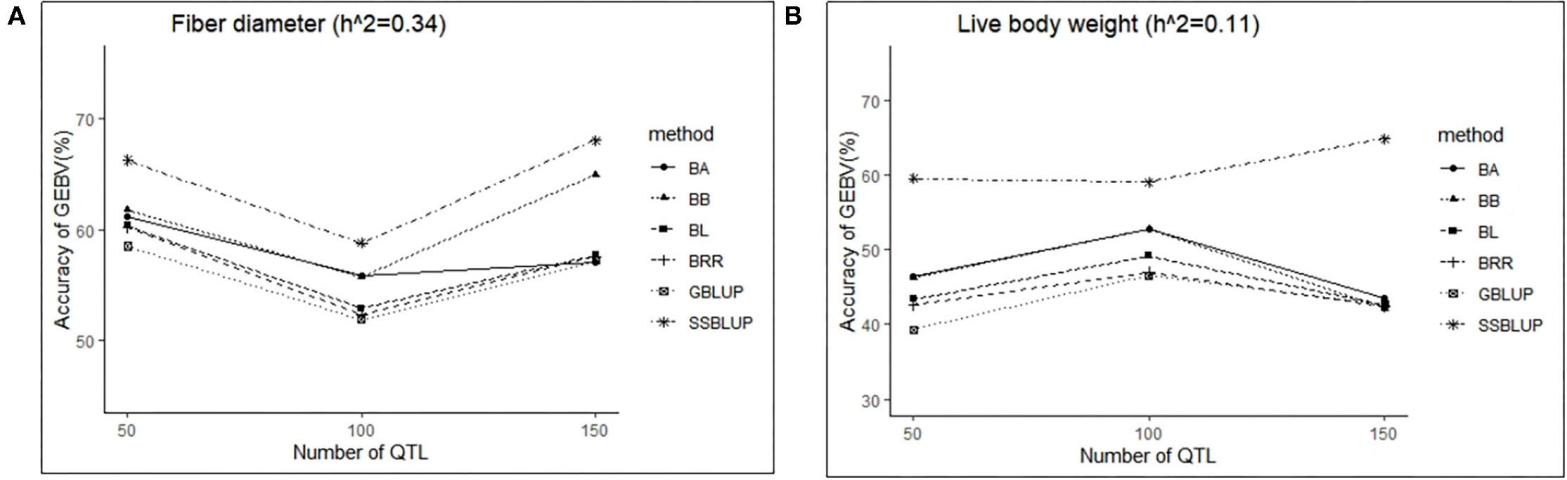
The change trends of accuracy of GEBV with increasing number of QTLs with GBLUP and Bayes methods.

In this study, three levels of QTLs (50, 100, and 150) were considered for analysis. For FD, the accuracy of GEBV in QTLs of 100 was significantly lower than the accuracy of GEBV in the other two levels, and the value was best when the number of QTLs was 50 (Table 6). Similarly, except for the ssGBLUP method, the trend of accuracy of GEBV with the number of QTLs showed that it decreased first and then increased (Figure 4A). For LBW, the accuracy of GEBV in QTLs of 100 was significantly higher than the accuracy of GEBV in the other two levels, and the value was up to 59.09% with the ssGBLUP method (Table 6). A first increasing and then decreasing trend was observed for the effect of the number of QTLs on the accuracy of GEBV (Figure 4B). The accuracy of GEBV with the number of QTLs in the GBLUP method was relatively lower than the accuracy of GEBV with the number of QTLs in the Bayesian methods. The accuracy of GEBV for medium- and low-heritability traits was better when the number of QTLs was 50 and 100, respectively.

**Table 6:** Accuracy of GEBV in three QTLs with heritability of fiber diameter and live body weight under different methods.

**Methods**	**Number of QTLs (*h*^**2**^ **=** 0.34)**			**Number of QTLs (*h*^**2**^ **=** 0.11)**		
	**50**	**100**	**150**	**50**	**100**	**150**
BA	0.6117 ± 0.0216^a^	0.5583 ± 0.0045^b^	0.5703 ± 0.0328^ab^	0.4636 ± 0.0251^a^	0.5269 ± 0.0340^a^	0.4360 ± 0.0062^a^
BB	0.6173 ± 0.0137^ab^	0.5568 ± 0.0262^b^	0.6493 ± 0.0175^a^	0.4619 ± 0.0250^ab^	0.5270 ± 0.0249^a^	0.4217 ± 0.0263^b^
BL	0.6032 ± 0.0156^a^	0.5290 ± 0.0191^b^	0.5767 ± 0.0266^ab^	0.4341 ± 0.0190^a^	0.4919 ± 0.0307^a^	0.4257 ± 0.0200^a^
BRR	0.6015 ± 0.0136^a^	0.5231 ± 0.0192^b^	0.5757 ± 0.0272^ab^	0.4259 ± 0.0215^a^	0.4696 ± 0.0304^a^	0.4241 ± 0.0148^a^
GBLUP	0.5850 ± 0.0121^a^	0.5190 ± 0.0165^a^	0.5718 ± 0.0221^a^	0.3932 ± 0.0462^a^	0.4658 ± 0.0206^a^	0.4264 ± 0.0041^a^
ssGBLUP	0.6623 ± 0.0309^a^	0.5877 ± 0.0344^a^	0.6799 ± 0.0240^a^	0.5954 ± 0.0193^a^	0.5909 ± 0.0071^a^	0.6489 ± 0.0154^a^

*BA, BayesA; BB, BayesB; BL, Bayesian Lasso; BRR, Bayesian Ridge Regression; GBLUP, Genomic Best Linear Unbiased Prediction; ssGBLUP, single step Best Linear Unbiased Prediction*.

a, b *represent significant differences. The difference is significant with different letters*.

### Effect of the Number of Males in the Reference Population on the Accuracy of GEBV

**Figure 5:**
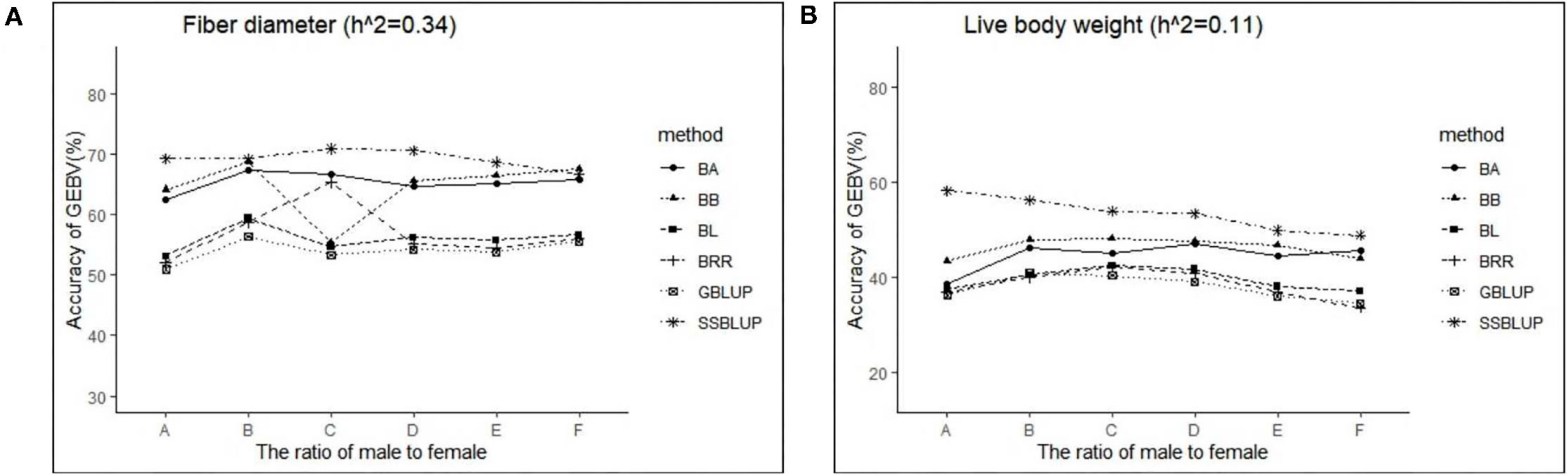
The change trends of accuracy of GEBV with increasing ratio of males to females with GBLUP and Bayes methods.

The variance analysis of the number of males in the reference population is presented in Tables 7, 8. For fiber diameter and live body weight, the number of males in the reference population had highly significant effect on accuracy of GEBV in goats (*P*< *0.01*). The multiple comparative analysis demonstrated that the higher the number of males size in the reference population, the more effective information can be provided, and the higher the accuracy of GEBV. Results from the effect of the reference population size have shown that 1,500 individuals were optimal to obtain significant genetic progress in both FD and LBW traits. Therefore, six groups were classified based on the ratio male (M) to female (F) in reference population (100 M + 1,400 F, 200 M + 1,300 F, 400 M + 1,100 F, 800 M + 700 F, 1,200 M + 300 F, 1,500 M). The effect of buck population size in the reference population on prediction accuracy was analyzed. The results indicated that the accuracy of GEBV was the highest when the buck population size was up to 400 (400 M + 1,100 F), which was significantly higher than the buck population size in other groups for FD (Table 9). The results indicated that the accuracy of GEBV was the highest when the buck population size was up to 100 (100 M + 1,400 F), which was significantly higher than the buck population size in other groups for LBW (Table 10). The accuracy values of GEBV in FD and LBW are 70.91 and 58.21%, respectively. In general, for FD and LBW, the trend of accuracy of GEBV with buck population size first increases and then remains stable (Figure 5). The trend for LBW was found to be basically consistent in each method. However, the trend for FD is irregular by BayesB method.

**Table 7:** Variance analysis of the ratio of male to female on the accuracy of GEBV for fiber diameter.

**Source**	**DF**	**SS**	**MS**	**F**	***P*-value**
The ratio of male to female	5	0.0196	0.0039	3.67	<0.01
Methods	5	0.3438	0.0688	64.32	<0.01
Error	97	0.1037	0.0011		
Corrected Total	107	0.4671			

*DF, degree of freedom; SS, St dev square; MF, mean square*.

**Table 8:** Variance analysis of the ratio of male to female on the accuracy of GEBV for live body weight.

**Source**	**DF**	**SS**	**MS**	**F**	***P*-value**
The ratio of male to female	5	0.0399	0.0080	5.50	<0.01
Methods	5	0.3263	0.0653	44.91	<0.01
Error	97	0.1409	0.0015		
Corrected Total	107	0.5071			

*DF, degree of freedom; SS, St dev square; MF, mean square*.

**Table 9:** Accuracy of GEBV in six levels ratio of male to female with heritability of fiber diameter under different models.

**Methods**	**The ratio of male to female (*h*^**2**^ **=** 0.34)**					
	**A 100 M + 1,400 F**	**B 200 M + 1,300 F**	**C 400 M + 1,100 F**	**D 800 M + 70 0F**	**E 1,200 M + 300 F**	**F 1,500 M**
BA	0.6239 ± 0.0147^b^	0.6740 ± 0.0117^a^	0.6676 ± 0.0139^ab^	0.6477 ± 0.0171^ab^	0.6518 ± 0.0240^ab^	0.6591 ± 0.0026^ab^
BB	0.6416 ± 0.0205^b^	0.6867 ± 0.0161^a^	0.5550 ± 0.0089c	0.6569 ± 0.0184^ab^	0.6647 ± 0.0265^ab^	0.6761 ± 0.0029^a^
BL	0.5312 ± 0.0352^b^	0.5939 ± 0.0132^a^	0.5472 ± 0.0115^b^	0.5620 ± 0.0359^ab^	0.5580 ± 0.0280^ab^	0.5669 ± 0.0049^ab^
BRR	0.5216 ± 0.0330^b^	0.5862 ± 0.0140^ab^	0.6535 ± 0.0155^a^	0.5523 ± 0.0368^b^	0.5449 ± 0.0273^b^	0.5611 ± 0.0030^b^
GBLUP	0.5096 ± 0.0366^b^	0.5635 ± 0.0110^a^	0.5330 ± 0.0115^ab^	0.5422 ± 0.0432^ab^	0.5377 ± 0.0386^ab^	0.5552 ± 0.0129^ab^
ssGBLUP	0.6930 ± 0.0423^ab^	0.6932 ± 0.0129^ab^	0.7091 ± 0.0136^a^	0.7063 ± 0.0136^ab^	0.6868 ± 0.0172^ab^	0.6671 ± 0.0228^b^

*BA, BayesA; BB, BayesB; BL, Bayesian Lasso; BRR, Bayesian Ridge Regression; GBLUP, Genomic Best Linear Unbiased Prediction; ssGBLUP, Single Step Genomic Best Linear Unbiased Prediction; M, male; F, female*.

a, b *represent significant differences. The difference is significant with different letters*.

**Table 10:** Accuracy of GEBV in six levels ratio of male to female with heritability of live body weight under different models.

**Methods**	**The ratio of male to female (*h*^**2**^ **=** 0.11)**					
	**A 100 M + 1,400 F**	**B 200 M + 1,300 F**	**C 400 M + 1,100,F**	**D 800 M + 700 F**	**E 1,200 M + 300 F**	**F 1,500 M**
BA	0.3866 ± 0.0185^b^	0.4628 ± 0.0524^ab^	0.4503 ± 0.0506^ab^	0.4698 ± 0.0243^a^	0.4460 ± 0.0590^ab^	0.4565 ± 0.0406^ab^
BB	0.4325 ± 0.0131^a^	0.4796 ± 0.0262^a^	0.4829 ± 0.0393^a^	0.4757 ± 0.0152^a^	0.4665 ± 0.0422^a^	0.4394 ± 0.0313^a^
BL	0.3732 ± 0.0137^a^	0.4042 ± 0.0287^a^	0.4248 ± 0.0372^a^	0.4171 ± 0.0366^a^	0.3803 ± 0.0610^a^	0.3705 ± 0.0271^a^
BRR	0.3687 ± 0.0144^ab^	0.3997 ± 0.0306^a^	0.4211 ± 0.0414^a^	0.4084 ± 0.0382^a^	0.3676 ± 0.0518^ab^	0.3363 ± 0.0138^b^
GBLUP	0.3619 ± 0.0249^a^	0.4090 ± 0.0383^a^	0.4033 ± 0.0423^a^	0.3906 ± 0.0363^a^	0.3605 ± 0.0598^a^	0.3455 ± 0.0436^a^
ssGBLUP	0.5821 ± 0.0384^a^	0.5622 ± 0.0263^ab^	0.5393 ± 0.0334^ab^	0.5340 ± 0.0338^ab^	0.4988 ± 0.0722^b^	0.4884 ± 0.0369^b^

*BA, BayesA; BB, BayesB; BL, Bayesian Lasso; BRR, Bayesian Ridge Regression; GBLUP, Genomic Best Linear Unbiased Prediction; ssGBLUP, Single Step Genomic Best Linear Unbiased Prediction; M, male; F, female*.

a, b * represent significant differences. The difference is significant with different letters*.

## Discussion

Previous studies illustrated that the genetic evaluation for fiber diameter in Inner Mongolia White Cashmere goats by using phenotype records of 1- and 2-year-olds could ensure the accuracy of EBV (38, 39). Considering the prohibitive cost and long duration of performance testing, genomic selection is gradually applied to animal breeding. The greatest advantage of genomic selection is that early selection can be achieved by collecting DNA samples at an early stage and genotyping individuals. The accuracy of genomic selection was influenced by many factors, such as SNP chip marker density, QTL numbers, genomic selection model, and so on. Therefore, it is necessary to discuss the factors affecting the accuracy of genomic selection in goats.

When more markers were distributed on the chromosome with a certain length, more favorable information could be provided. Detection of markers that are in linkage disequilibrium with QTLs is easier. Generally, a higher accuracy of genomic selection would be obtained with greater marker density. However, when marker density reaches a certain number, the accuracy of genomic selection will not increase significantly to some extent, or the growth rate will slow down. Increasing marker density from 24 to 728 K SNPs resulted in a small increase in the accuracy of GEBV in three cow breeds with the GBLUP method, and the values of 24 and 728 K were 0.22 and 0.24, respectively (40). The cost of genotyping is closely related to the marker density panel. However, some studies have shown that the accuracy of GEBV by using genotype information with high-density chips is higher than the accuracy of GEBV by using genotype information with low-density chips (41, 42). The excessive cost of genotyping limited the realization of genomic selection in low-income species, such as goats. In production practice, the goal of breeders is to achieve equivalent effects using low-density SNP chips, as well as high-density chips, which can reduce sequence cost and improve the accuracy of selection. The results obtained from our study were similar to the results from previous reports. The prediction accuracy increased with increasing marker density chips. For both traits, the medium marker density chip was most effective in genomic selection of goats. Solberg et al. reported that the accuracy of genome estimation breeding value increased significantly with increasing marker density by simulation (43). The linkage disequilibrium between adjacent SNP markers was positively correlated with the accuracy of genomic breeding value. The degree of linkage disequilibrium depends on the marker density (44).

Many studies have reported that the influence of reference population size has an effect on genomic selection accuracy. Generally, a higher GEBV prediction accuracy was obtained with a larger reference population size. When the population size is small, the genomic relationship matrix cannot hold enough genomic information (independent chromosome segments). Therefore, the accuracy can be lower with the smaller size. Therefore, the number of individuals in the reference group should be increased as much as possible when genomic selection is performed. However, the optimal reference population size must be considered because of high sequencing costs. Zengting Liu et al. estimated the genomic breeding value of milk yield in dairy cattle, which indicated that the additive effect variance increased five times when the size of the reference population increased from 734 to 5,025 (45). In our study, when the reference population increased from 500 to 3,000, the accuracy of GEBV in the medium and low heritability traits increased by 15 and 20%, respectively. (46) evaluated the effect of reference population size on genomic selection in dairy goats.

The population size was demonstrated to have an important effect on GEBV accuracies, from 2 to 31% with the reference population from 1,966 to 2,651 (46). However, Moser et al. reported the genomic selection of milk protein in Holstein cattle, which explained that the GEBV accuracy showed no obvious change for the reference population from 1,239 to 1,822 (47). All these results indicated that the accuracy of genomic selection can be effectively ensured when the reference population reaches a certain level.

Generally, the genetic variance was assumed to be one regardless of the number of QTLs. When the number of QTLs is large, the variance proportion of each QTL decreases. That is, the contribution of each QTL to phenotypic value decreases, and the probability of the effect (or variance) of each QTL correctly estimated will be relatively low, which will lead to an increase in deviation and a decrease in the accuracy of GEBV. When the number of QTLs is small, it will be hard to estimate the additive genetic variance or heritability, assuming no polygenic effects, especially for a small data size. The results from our study were consistent with this point of view. Zhang et al. reported that the accuracy of GEBV decreased with the increase in the number of QTLs from 50 to 1,000. The BayesB methods seemed to be more sensitive to the number of QTLs than the GBLUP method (48), which is similar to our study. Daetwyler et al. compared the genomic selection accuracy between the GBLUP and BayesB methods, which demonstrated that the prediction accuracy with the BayesB method was greatest at low NQTLs and decreased with increasing NQTLs. However, as N_QTL_ increased, the difference between the two methods decreased, and eventually, both approaches achieved very similar accuracy (49).

Sex chromosomes play a significant role in key evolutionary processes such as speciation and adaptation (50). The male to female ratio could affect accuracy of GEBV because it changes the effective population size as well as LD. Previous studies have shown that the accuracy of genomic selection can be improved by increasing male size in reference populations. Avendano (51) reported that the accuracy of GEBV decreased with an increase in the ratio of males to females in chickens, and the values increased from 0.33 to 0.5 (51). Céline (46) showed that the GEBV accuracies with increasing male size in the reference population were not improved (46). Our results demonstrated that the accuracy of GEBV showed no significant change when the buck population size was up to 200. The obvious results in different studies were explained by breeds, methods, and other factors.

Genomic selection is an effective way to accelerate the genetic improvement of traits with low heritability and unmeasurable traits. In our study, two traits with medium and low heritability were used for analysis. The results showed that the GEBV accuracy for medium heritability traits was higher than the GEBV accuracy for low heritability traits, which is consistent with previous reports. Villumsen et al. evaluated the effects of heritability on genomic estimated breeding value. The accuracy of the genomic estimated breeding value was found to increase from 0.69 to 0.86 when heritability increased from 0.02 to 0.30 (20). Zhang et al. reported the accuracies of GEBV by different methods and various heritability traits (48). By decreasing the heritability from 0.90 to 0.05, the prediction accuracies with all methods decreased significantly. The accuracy of GEBV for heritability of 0.1 and 0.3 was slightly higher than the accuracy of GEBV in our study, with heritability traits of 0.11 and 0.34.

Many methods, including GBLUP, ssGBLUP, and Bayes methods, have been used to perform genomic selection in plants and animals. To some extent, the methods affected the accuracy of the prediction accuracy. Gao et al. compared the efficiency of four Bayesian models and the GBLUP model on the GEBV accuracy, which indicated that the superiority of the Bayesian models over the GBLUP model was more profound (52). Sun et al. compared the accuracy of GEBV obtained by BayesB, RRBLUP, and GBLUP using simulated datasets. The prediction accuracy with BayesB was found to be higher than the prediction accuracy with RRBLUP and GBLUP. There were no significant differences among the methods (53). Clark et al. (21) compared the impact of ABLUP, GBLUP, and BayesB on the accuracy of genetic evaluation. For the ABLUP method, the numeric relationship matrix (NRM) was calculated by pedigree. The results showed that the BayesB method would be more accurate if important QTLs had an effect on the traits. However, Clark et al. reported that Bayes and GBLUP methods had similar prediction accuracy when each QTL had a small effect (21).The ssGBLUP method used both genotype information and pedigree information to construct the relationship matrix when GEBV was obtained, which is an ideal alternative for genomic genetic evaluation compared with other methods. Lourenco et al. reported that predictive ability of genomic EBV for growth traits and calving ease when using single-step genomic BLUP (ssGBLUP) in Angus cattle was higher than that in using BLUP (54). Teissier et al. illustrated that the accuracy of GEBV for milk production traits, udder type traits, and somatic cell scores in French dairy goats was higher than that using other methods. Similarly, the accuracy of GEBV in ssGBLUP for FD and LBW was higher than that with other methods in our study (55). In addition, the computation efficiency for ssGBLUP was also relatively good by comparing with the Bayes methods. Therefore, the ssGBLUP method was suggested to perform genomic selection in goats.

## Conclusions

All the results in this study determined the optional level of factors influencing the accuracy of genomic estimated breeding value of FD and LBW in goats. The medium marker density panels were designed for genotyping, which can effectively ensure the accuracy of genomic selection of goats. When the reference population size was up to 1,500, genomic selection of cashmere and meat goats was performed. The accuracy of GEBV for FD and LBW was better when the number of QTLs was 50 and 100, respectively, indicating that both traits were controlled by minor genes. Meanwhile, the accuracy of GEBV was discovered to be good when the buck population size in the reference population was up to 200. All these factors will make a reasonable judgement on the factors affecting genomic selection and lay a foundation for the subsequent realization of genomic selection in cashmere and meat goat breeding.

## Data Availability Statement

The original contributions presented in the study are included in the article/supplementary material, further inquiries can be directed to the corresponding author/s.

## Author Contributions

ZhiyW, RS, and XiaocY conceived of and coordinated the study. YZ, ZhixW, ZL, JZ, RW, CD, and JL helped in conceive of the study. GG, FW, LZ, and QL simulated the data.TZ, LL, YY, GY, YH, HM, HL, YL, WL, and XiaomY analyzed the data. XiaocY and ZhiyW wrote the manuscript. All authors read and approved the final manuscript.

## Funding

The authors are grateful for the grants supported by Natural Science for Youth Foundation (31702086), IMAR (Inner Mongolia Autonomous Region) Natural Science Foundation (2019MS03070), Inner Mongolia Autonomous Region Science and Technology Research Project (2021GG0086), Inner Mongolia Agricultural University for second levels of outstanding doctorate (NDYB2016-05), Science and technology major project of Inner Mongolia Autonomous Region (2021ZD0012), China Agriculture Research System of MOF and MARA (No. CARS-39), Scientific Research Projects of Institutions of Higher Learning in Inner Mongolia Autonomous Region (NJZY19104), and IMAR (Inner Mongolia Autonomous Region) Natural Science Foundation (2019MS08121).

## Conflict of Interest

TZ is only employed by the company Inner Mongolia Bigvet Co., Ltd. The remaining authors declare that the research was conducted in the absence of any commercial or financial relationships that could be construed as a potential conflict of interest.

## Publisher's Note

All claims expressed in this article are solely those of the authors and do not necessarily represent those of their affiliated organizations, or those of the publisher, the editors and the reviewers. Any product that may be evaluated in this article, or claim that may be made by its manufacturer, is not guaranteed or endorsed by the publisher.
